# *Afrostyrax lepidophyllus extracts* exhibit in vitro free radical scavenging, antioxidant potential and protective properties against liver enzymes ion mediated oxidative damage

**DOI:** 10.1186/s13104-015-1304-8

**Published:** 2015-08-12

**Authors:** Bruno Moukette Moukette, Constant Anatole Pieme, Prosper Cabral Nya Biapa, Vicky Jocelyne Ama Moor, Eustace Berinyuy, Jeanne Yonkeu Ngogang

**Affiliations:** Laboratory of Biochemistry, Department of Physiological Sciences and Biochemistry, Faculty of Medicine and Biomedical Sciences, University of Yaoundé I, PO Box 1364, Yaoundé, Cameroon; Department of Biochemistry, Faculty of Science, University of Dschang, PO Box 67, Dschang, Cameroon

**Keywords:** *Afrostyrax lepidophyllus*, Antioxidant, Spice, Non-timber forest product, Polyphenols, Scavenging activity

## Abstract

**Background:**

Several studies described the phytochemical constituents of plants in relation with the free radical scavenging property and inhibition of lipid peroxidation. This study investigated the in vitro antioxidant property, and the protective effects of ethanolic and aqueous ethanol extract of the leaves and barks of *Afrostyrax lepidophyllus* (Huaceae) against ion mediated oxidative damages.

**Methods:**

Four extracts (ethanol and aqueous-ethanol) from the leaves and barks of *A. lepidophyllus* were used in this study. The total phenols content, the antiradical and antioxidant properties were determined using standard colorimetric methods.

**Results:**

The plant extracts had a significant scavenging potential on the 2,2-diphenyl-1-picrylhydrazyl (DPPH), hydroxyl (OH), nitrite oxide (NO) and 2,2-azinobis (3-ethylbenzthiazoline)-6-sulfonic acid (ABTS) radicals with the IC_50_ varied between 47 and 200 µg/mL depending on the part of plant and the type of extract. The ethanol extract of *A. lepidophyllus* bark (GEE) showed the highest polyphenolic (35.33 ± 0.29) and flavonoid (12.00 ± 0.14) content. All the tested extracts demonstrated a high protective potential with the increased of superoxide dismutase, catalase and peroxidase activities.

**Conclusion:**

*Afrostyrax lepidophyllus* extracts exhibited higher antioxidant potential and significant protective potential on liver enzymes.

**Electronic supplementary material:**

The online version of this article (doi:10.1186/s13104-015-1304-8) contains supplementary material, which is available to authorized users.

## Background

Oxidative stress is a disturbance between the pro-oxidant/antioxidant balance in favor of the former, leading to cell damage [1, 2]. Reactive oxygen or nitrogen species (ROS, RNS) are important free radicals involved in the human function. They play important physiological role such as immuno-competence, apoptosis, vascular tone, hormonal regulation, signal transduction, transcription factors, defense genes and adaptive responses to enzymes [3–6]. In higher concentration, they can be harmful [7, 8]. Recent studies demonstrated that they contribute to the aging process and the initiation or the development of a wide range of pathologies [5, 9]. ROS can induce several mutations in DNA such as base modifications, rearrangement of DNA sequences, miscoding of DNA lesions, gene duplications, and activation of oncogenes leading to the initiation and propagation of tumor growth, angiogenesis and metastasis [9, 10]. However, cells have a comprehensive array of antioxidant defense mechanisms to reduce free radical formation or limit their damaging effects [11]. This defense system consists of enzymatic [superoxide dismutase (SOD), catalase and peroxidase] and non-enzymatic antioxidant defenses which counteract the harmful effects of free radicals. The non-enzymatic is consisting of reduced glutathione and food derived antioxidants such as vitamins, polyphenols and some minerals. Nowadays, the use of natural antioxidants from plant origin is receiving great attention. Several studies demonstrated the antioxidant activities, phytochemical constituents of plants and spices as well as their inhibitors effects against lipid peroxidation [11–14].

*Afrostyrax lepidophyllus* (bush onion) is a non-timber forest product from the Huaceae family found in the green forest in Ghana, Cameroun and Republic of Congo. The leaves and fruits of *A. lepidophyllus* have a strongly offensive smell of onion or garlic. This fruit is used as spice in the traditional African cuisine. In folk medicine, the root and bark decoctions are drunk as anthelmintic against vomiting or as enema against urinary infections [15, 16]. Crushed of young leaves or leaves decoction are used as bath seat against hemorrhoids frigidity for women, as laxative against urinary infections, mouthwash against caries and toothache [15, 16]. The inner barks are applied locally after incisions in case of snake bites [17]. The essential oils from the seeds of the plant demonstrated cytotoxic and antimicrobial activities [18]. In the light of these informations it is obvious that a large number of scientific data are still needed in order to promote the use of *A. lepidophyllus* for the management of health problem. The main objective of this study was to investigate the in vitro antioxidant, free radical scavenging properties, and the protective effects of ethanol and aqueous ethanol extract from leaves and barks of *A. lepidophyllus* against oxidative mediated liver injuries.

## Methods

### Plant material

The leaves and barks of *A. lepidophyllus* were collected at the Kala Mountain in the center region of Cameroon. They were authenticated by Mr NANA, a botanist of the National Herbarium of Cameroon, in comparison to the voucher specimens (39020 HNC).

### Preparation of plant extracts

The collected leaves and barks were dried at ambient temperature and crushed. The powders were then macerated in the ratio of 1:10 (w/v) for 48 h in ethanol for the ethanol extract and in a mixture of water + ethanol (30/70); pH = 3 for the aqueous ethanol extract. The mixtures were then filtered using a Buchner funnel and Whatman No. 1 filter paper. This process was repeated once on the residue. The filtrate was concentrated using a rotavapor and the solution was dried in the oven at 55°C for 2 days. Each crude extract obtained was labelled using the following codes: GEE for the ethanol extract of *A. lepidophyllus* bark; GFE for the ethanol extract of *A. lepidophyllus* leaves, GEH for the aqueous ethanol extract of *A. lepidophyllus* bark, GFH for the aqueous ethanol extract of *A. lepidophyllus* leaves. The different samples were then kept at 4°C. Prior to the experimentation, the solutions of the four plant extracts were dissolved using ethanol different dilutions (25, 50, 75, 150, 300 µg/mL) of each.

### Determination of the free radical scavenging and antioxidant properties

#### Scavenging activity of DPPH radical

The DPPH assay measures the free radical scavenging capacity of the extracts under investigation [19]. Each of the diluted extracts (3 mL) were put in the test tube and 1 mL of a methanol solution of DPPH (0.1 mM) was added. The mixture was kept in the dark at room temperature for 30 min and absorbance was measured at 517 nm against a blank. The same procedure was used for the vitamin C (1 mg/mL) used as standard. The following equation was used to determine the percentage of the radical scavenging activity of each extract.

$$Scavenging\;effect\;(\%) = 100 \times (A_{o} - A_{s})/A_{o}$$

where A_o_ is the absorbance of the blank and A_s_ the absorbance of the sample.

#### Scavenging effect of the ABTS^+^ radical

The ABTS assay was based on a previously described method [20] with slight modifications. ABTS radical cation (ABTS^+^) was produced by the reaction of a 7 mM ABTS solution with potassium persulphate (2.45 mM). The ABTS^+^ solution was diluted with ethanol to an absorbance of 0.70 ± 0.05 at 734 nm. The mixture was stored in the dark at room temperature for 12 h before used. After addition of 25 µL of extract sample or vitamin C used as standard to 2 mL of diluted ABTS^+^ solution, absorbance was measured at 734 nm after exactly 6 min. The decrease in absorption was used for calculating scavenging effect values. The following equation was used to determine the percentage of the radical scavenging activity of each extract.

$$Scavenging\;effect\;(\%) = 100 \times (A_{o} - A_{s})/A_{o}$$

where A_o_ is the absorbance of the blank; A_s_ is the absorbance of the sample.

#### Nitric oxide scavenging activity

Nitric oxide scavenging activity was determined according to the Griess Illosvoy reaction [21]. The reaction mixture contained 2 mL of sodium nitroprusside (10 mM) in 0.5 mL phosphate buffer (0.5 M; pH 7.4). Various concentrations (25, 50, 75, 150, 300 µg/mL) of the extracts (0.5 mL) were added in a final volume of 3 mL. After incubation for 60 min at 37°C, Griess reagent [N-(1-Naphthyl) ethylenediamine (0.1%) and sulphanilic acid (1%) in H_3_PO_4_ (5%)] was added. The pink chromophore generated during diazotization of nitrite ions with sulphanilamide and subsequent coupling with N-(1-Naphthyl) ethylenediamine was measured spectrophotometrically at 540 nm. Ascorbic acid was used as a positive control. The scavenging ability (%) of the nitric oxide was calculated using the formula:

$$Scavenging\;effect\;(\%) = 100 \times (A_{o} - A_{s})/A_{o}$$

where A_o_ is the absorbance of the blank and A_s_ the absorbance of the sample.

#### Hydroxyl radical scavenging activity

The scavenging activity of the extracts on hydroxyl radical was measured according to a previously described method [22]. To 1.5 mL of each diluted extract, 60 μL of FeCl_3_ (1 mM), 90 μL of 1,10-phenanthroline (1 mM), 2.4 mL of phosphate buffer (0.2 M; pH 7.8) and 150 μL of H_2_O_2_ (0.17 M) were added respectively. The mixture was then homogenized using a vortex and incubated at room temperature for 5 min. The absorbance was read at 560 nm against the blank. The percentage of the radical scavenging activity of each extract was calculated from the equation below:

$$Scavenging\;effect\;(\%) = 100 \times (A_{o} - A_{s})/A_{o}$$

where A_o_ is the absorbance of the blank and A_s_ the absorbance of the sample.

### Total antioxidant activity by ferric reducing antioxidant power assay (FRAP)

The FRAP was determined using a previously described method [23] with slight modifications. The fresh FRAP reagent consisted of 500 mL of acetate buffer (300 mM; pH 3.6), 50 mL of 2,4,6-Tris(2-pyridyl)-*s*-triazine (TPTZ) (10 mM), and 50 mL of FeCl_3_·6H_2_O (50 mM). The colorimetric measurement was performed at 593 nm and the measurement was monitored up to 12 min on 75 μL of each extract and 2 mL of FRAP reagent. The vitamin C was used to draw a standard curve and the butylated hydroxy toluene (BHT) was used for the comparison. The results were expressed as mg equivalent vitamin C/g of dried extract (mg eq VitC/g DE).

### Phosphomolybdenum antioxidative power (PAP)

The total antioxidant activity of the extracts was evaluated by green phosphomolybdenum complex according to a method described [24]. An aliquot of 10 μL of the extract solution was mixed with 1 mL of reagent solution (0.6 M sulphuric acid, 28 mM sodium phosphate and 4 mM ammonium molybdate) in a micro centrifuge tube. The tubes were incubated in a dry thermal bath at 95°C for 90 min. After cooling, the absorbance of the mixture was measured at 695 nm against a blank. The vitamin C was used as reference to draw the standard curve and BHT (1 mg/mL) was used for the comparison. The reducing capacities of the analysed extracts were expressed as mg of ascorbic acid equivalents/g of dried extract (mg eq AS/g DE).

### Reducing power assay

The reducing power of the extracts was determined by a method described [25]. Different concentrations of extracts in 1 mL of distilled water were mixed with 2.5 mL of phosphate buffer (0.2 M, pH 6.6) and 2.5 mL of potassium ferrocyanide (1%). The mixtures were incubated at 50°C for 20 min. Aliquots 2.5 mL of trichloroacetic acid (10%) were added to the mixtures and centrifuged at 3,000 rpm for 10 min. The supernatant of the solution (2.5 mL) was mixed with 2.5 mL of distilled water and 0.5 mL of FeCl_3_ (0.1%). The absorbance was measured at 700 nm.

### Total phenol determination

The total phenol content was determined by the Folin–Ciocalteu method [26]. The reaction mixture contained 200 μL of extract, 800 μL of freshly prepared diluted Folin Ciocalteu reagent and 2 mL of sodium carbonate (7.5%). The final mixture was diluted to 7 mL with deionized water and kept in the dark at ambient conditions for 2 h to complete the reaction. The absorbance was measured at 765 nm. Caffeic acid was used as standard and the results were expressed as mg caffeic acid/g dried extract (mg CA/g DE).

### Determination of total flavonoid content

Total flavonoid content was determined using aluminium chloride (AlCl_3_) according to a known method [27] using quercetin as a standard. A volume of 0.1 mL of spice extract was added to 0.3 mL distilled water followed by 0.03 mL of NaNO_2_ (5%). After 5 min at 25°C, 0.03 mL of AlCl_3_ (10%) was added. After a further 5 min, the reaction mixture was mixed with 0.2 mL of 1 mM NaOH. Finally, the reaction mixture was diluted to 1 mL with water and the absorbance was measured at 510 nm. The results were expressed as mg quercetin (QE)/g of dried extract (QE/g dried ext).

### Determination of total flavonols

Total flavonols in the plant extracts were estimated using a known method [28] with modifications. To 2.0 mL of sample, 2.0 mL of 2% of ethanolic solution of AlCl_3_ and 3.0 mL (50 g/L) sodium acetate solutions were added. After 2.5 h of incubation at 20°C, the absorbance was read at 440 nm. The results were expressed as quercetin equivalent (mg/g) dried extract (QE/g dried ext).

### Protective properties of the plant against oxidative damage

#### Preparation of liver homogenate

The liver was isolated from three normal albino Wistar rats. The organs were weighed and 10% (w/v) homogenate was prepared in phosphate buffer (0.1 M, pH 7.4 having 0.15 M KCl) using the homogenizer at 4°C [29]. The homogenate was centrifuged at 3,000 rpm for 15 min and the clear cell-free supernatant obtained was used for the study.

#### Preparation of the pro-oxidative solution

The oxidant solution was prepared, directly before its utilization by adding a solution of ferric chloride 100 mM to H_2_O_2_ 0.50% prepared in phosphate buffer (0.1 M, pH 7.4). This solution was used for the investigation of the protective assays on liver enzymes.

#### Total protein content

The total protein content of the mixture of liver was measured according to the protein kit supplier method (Human Kit-Hu102536, Boehringer Ingelheim, Germany). This result was used to express the activities of the different enzymes per gram of organs.

#### In vitro lipid peroxidation assay

Lipid peroxidation assay was performed by a formerly described protocol [30]. Phosphate buffer 0.58 mL (0.1 M; PH 7.4), 200 μL sample, 200 μL liver homogenate and 20 μL ferric chloride (100 mM) were combined to form mixture a which was placed in a shaking water bath for 1 h at 37°C. The reaction was terminated by adding 1 mL trichloroacetic acid (TCA) (10%), thiobarbituric acid (TBA) 1 mL (0.67%) to all the tubes which were placed in boiling water bath for 20 min. Then test tubes were shifted to crushed ice bath and were centrifuged at 3,000 rpm for 10 min. Absorbance of the supernatant was checked at 535 nm and was calculated as nM of MDA tissue using molar extinction coefficient of 1.56 × 10^5^/M cm.

#### Determination of peroxidase activity

Peroxidase activity was determined by the peroxidase kit (CAS Number 7722-84-1, Sigma Aldrich) supplier with modifications. A solution containing the mixture of 1 mL of the oxidant solution (FeCl_3_, 100 mM) and extract or vitamin C (standard) for a final concentration of 100 µg/mL was incubated for 1 h in a water bath at 37°C. An aliquot of PBS (0.1 mL), hydrogen peroxide (50 μL), and pyrogallol solution (110 μL) were added to distilled water (625 μL) that was earlier dispensed into an Eppendorf tube. The plant extract (75 μL) from the mixture was thereafter added. For the blank, the control oxidant solution and the vitamin C as standard, the same reagents were used, except the extract which was replaced by distilled water (75 μL). The reaction was mixed and incubated for at least 10 min. The solution containing 100 mM, pH 6.0 PBS (40 μL) and 0.002% (v/v) diluted liver homogenate (40 μL) were added to the blank and test mixtures respectively. These were mixed, and the increase in absorbance at 420 nm was measured at every 30 s for 3 min using a spectrophotometer (BioMate 3S UV–Visible, Thermo Scientific™ Manufacturer, Wohlen, Switzerland). One unit of peroxidase was defined as the change in absorbance/s/mg of protein at 420 nm using molar extinction coefficient of 12/M cm.

#### Determination of catalase activity

Prior to the test, a solution containing a mixture of 1 mL of total volume of the oxidant solution and extract or vitamin C (standard) for a final concentration of 100 µg/mL was incubated for 1 h in a water bath at 37°C. The catalase activity of liver homogenate was assayed as previously described with modifications [31]. An aliquot of hydrogen peroxide (0.8 mL) was dispensed into an Eppendorf tube. Phosphate buffer (1.0 mL), extracted sample/vitamin C/oxidant solution (75 μL) and (0.002% v/v) diluted homogenate (125 μL) were added. The reaction mixture (0.5 mL) was dispensed into 5% dichromate reagent (1.0 mL) and vigorously shaken. The mixture was heated in a Clifton water bath for 10 min, and allowed to cool. The absorbance at 570 nm was taken using spectrophotometer (BioMate 3S UV–Visible, Thermo Scientific™ Manufacturer, Wohlen, Switzerland). The absorbance obtained was extrapolated from the following standard curve y = 0.0028x + 0.0132. The catalase activity was thereafter expressed as Unit/min/mg of protein (UI/mg Prot.)

$$CAT(({\text{unit}})/{\text{mg}}\;{\text{protein}}) = (Abs/\hbox{min} \times 30000\;{\text{units}})/(40\;{\text{cm/M}} \times {\text{mg}}\;{\text{protein}}) \times df$$

where df is the dilution factor, Abs the absorbance.

#### Superoxide dismutase (SOD) activity

The measurement of total SOD activity was performed according to the Misra and Fridovich method with some slight modifications [32]. The principle of this method is based on the inhibition of epinephrine autoxidation. Distilled water (0.2 mL) and 2.5 mL sodium carbonate buffer 0.05 M, pH 10.2 were added to the 0.3 mL buffered epinephrine to initiate the reaction. The absorbance at 480 nm was read for 150 s at 30 s intervals against a blank made up of 2.5 mL buffer, 0.3 mL epinephrine and 0.2 mL distilled water. The following equation allowed the calculation of the SOD activity:

$$SOD({\text{unit/mg}}\;{\text{protein}}) = SOD({\text{units/mL/min}})/protein({\text{mg/mL}}) \times df$$

where df is the dilution factor,

The SOD activity was there after expressed as unit/min/mg of protein (UI/mg Prot.)

### Statistical analysis

The results were presented as mean ± SEM of triplicate assays. Analyses of data was conducted using one-way analysis of variance (ANOVA) followed by Kruskal–Wallis test and Dunnett’s multiple test (SPSS program version 18.0 for Windows, IBM Corporation, New York, NY, USA). The Log probit was used to determinate the IC_50_ using the software XLstat version 7 (Addinsoft, New York, NY, USA) were used to achieve the Pearson Correlation Analysis (PCA). The differences were considered as significant at *P* < 0.05.

## Results

### Scavenging potential of different radicals and polyphenols content

**Fig. 1:**
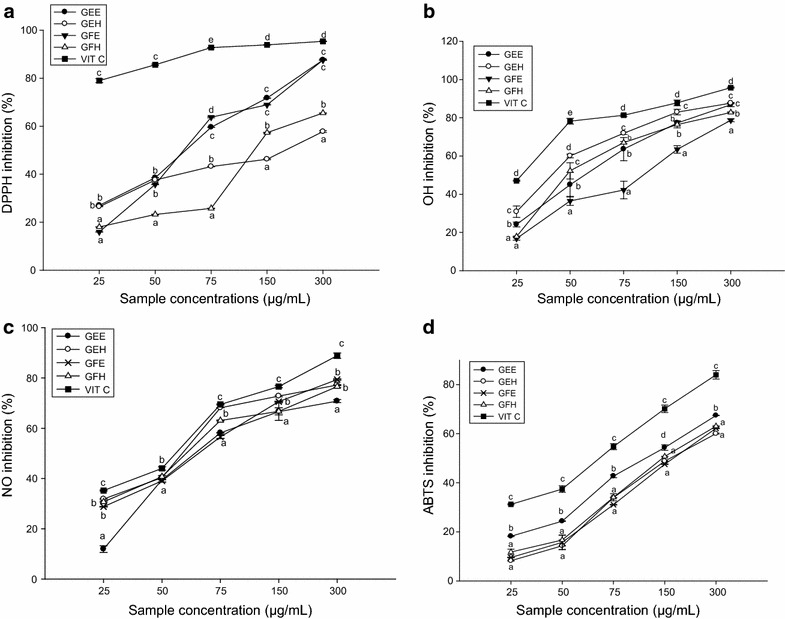
Scavenging potential of the different plant extracts; **a** DPPH^∙^; **b** OH^∙^; **c** NO^∙^; **d** ABTS^∙^. Values are expressed as mean ± SD of three replicates. In the same colon the values affected with *different letter* are significantly different at p < 0.05. *GEE* ethanolic extract of bark of *A. lepidophyllus*, *GFE* ethanolic extract of leaves of *A. lepidophyllus*, *GEH* aqueous ethanol extract of bark of *A. lepidophyllus*, *GFH* aqueous ethanol extract of leaves of *A. lepidophyllus*, *VIT C* vitamin C.

The effects of extracts on different free radicals is representing in the Fig. 1. All the extracts demonstrated significant scavenging activities which varied depending on the part of the plant and the solvent used. For the DPPH radical, the extract GEE and GFE showed a higher inhibitory potential while GEH exhibited the lowest inhibitory potential with a percentage of 57.36 ± 2.51% at the highest concentration (Fig. 1a) while for the hydroxyl radical, the highest values varied from 78.73 ± 0.74% (GFE) to 87.66 ± 0.28% (GEH) at the highest concentration (Fig. 1b). The results nitric oxide scavenging potential of the different extract samples represented in the Fig. 1c showed that the inhibitory potential of the different samples increased with the concentration. GEE extract showed the lower potential at the concentration 25 µg/mL (11.89 ± 1.33%) while GEH and GFH extracts exhibited the highest inhibitory potential (Fig. 1c). Regarding the ABTS radical, all the tested samples demonstrated a scavenging potential (Fig. 1d). Among them, GEE showed the highest inhibitory percentage at the different concentrations varying from 18.21 ± 0.14% at the concentration of 25 µg/mL to 67.44 ± 0.10% at the concentration of 300 µg/mL. The values of IC_50_ of different the radicals tested represented in the Additional file 1: Table S1. These results showed that the IC_50_ of all the scavenged radicals varied from 47.77 to 200 µg/mL depending on the type of radicals, the plant extracts or the solvent used. These values are higher compared to the vitamin C. The lowest IC_50_ value was 47.77 ± 1.09 and 63.08 ± 1.44 µg/mL (GEH) for hydroxyl radical and nitrite radicals respectively, 64.66 ± 0.02 µg/mL (GEE) and 94.95 ± 2.31 µg/mL (GEH) respectively for DPPH and ABTS radicals. The levels of polyphenol, flavonoids and flavonols of the different plant extract represented in the Additional file 2: Table S2 demonstrated that the GEE (35.33 ± 0.29 [(CAE)/g dried ext.] and GFE [34.11 ± 0.87 (CAE)/g dried ext] have the highest polyphenol contents compared to other extracts. The highest concentration of flavonoid (12.00 ± 0.14 QE/g dried ext.) was identified in the GEE while the GFE showed the highest flavonol content (3.50 ± 0.40 QE/g dried ext). The significant and positive correlation between flavonoids and DPPH, NO, ABTS and polyphenol content was noted (Additional file 3: Table S3).

### Reductive activities of the different extract samples

**Fig. 2:**
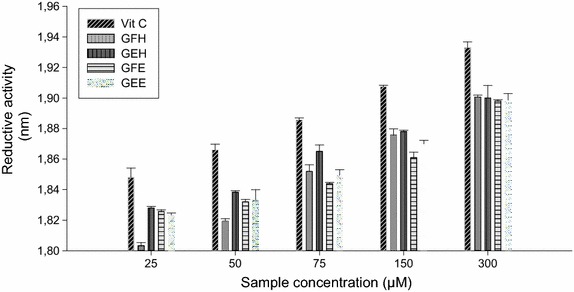
Reductive activities of the different plant extracts. Values are expressed as mean ± SD of three replicates. In the same colon the values affected with *different letter* are significantly different at p < 0.05. *GEE* ethanolic extract of bark of *A. lepidophyllus*, *GFE* ethanolic extract of leaves of *A. lepidophyllus*, *GEH* aqueous ethanol extract of bark of *A. lepidophyllus*, *GFH* aqueous ethanol extract of leaves of *A. lepidophyllus*, *VIT C* vitamin C.

The reductive activities of the different plant extracts presented in the Fig. 2 showed that the vitamin C exhibited the highest reductive activity at all the concentrations used. Among the tested plants, at the concentrations of 75 and 150 µg/mL, GFH and GEH showed the highest reductive potentials.

### Antioxidant activities of the extracts using FRAP and PAP methods

**Fig. 3:**
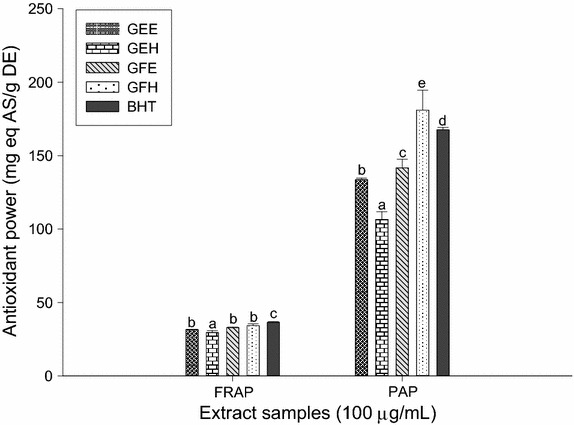
Antioxidant activities of the different plant extracts using FRAP and phosphomolybdate antioxidant power. Values are expressed as mean ± SD of three replicates. In the same colon the values affected with *different letter* are significantly different at p < 0.05. *GEE* ethanolic extract of bark of *A. lepidophyllus*, *GFE* ethanolic extract of leaves of *A. lepidophyllus*, *GEH* aqueous ethanol extract of bark of *A. lepidophyllus*, *GFH* aqueous ethanol extract of leaves of *A. lepidophyllus*, *VIT C* vitamin C.

The FRAP antioxidant activities and the phosphomolybdenum antioxidant power (PAP) of the different plant extracts are shown in the Fig. 3. We noted that GFH extract had the higher antioxidant potentials using FRAP and PAP methods compared to the other extracts. Concerning the phosphomolybdenum antioxidant power (PAP), the antioxidant activity of GFH was lower compared to that of BHT used as the standard at the same concentration.

### Protective property of extract samples against lipid peroxidation

**Fig. 4:**
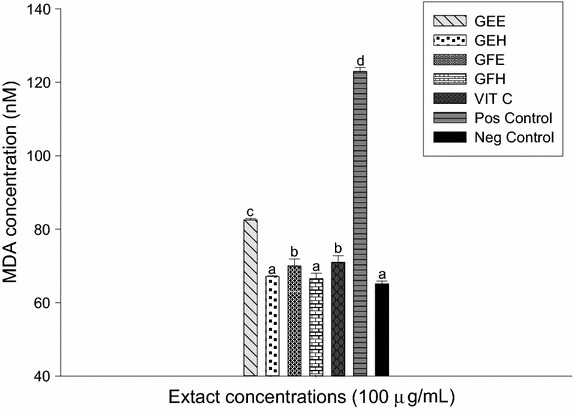
Protective properties of plant extracts against lipid peroxidation. Values are expressed as mean ± SD of three replicates. In the same colon the values affected with *different letter* are significantly different at p < 0.05. *GEE* ethanolic extract of bark of *A. lepidophyllus*, *GFE* ethanolic extract of leaves of *A. lepidophyllus*, *GEH* aqueous ethanol extract of bark of *A. lepidophyllus*, *GFH* aqueous ethanol extract of leaves of *A. lepidophyllus*, *VIT C* vitamin C, *Pos Control* oxidant (positive) control, *Neg Control* normal (negative) control.

The Fig. 4 showed the results of the effect of the extracts on lipid peroxidation. As we can find in this figure, the level of MDA was significantly higher (122.95 ± 088 µmol/L) in the group with oxidant (positive control) compared to the group without oxidant (negative control) 65.13 ± 3.87 µmol/L. GEH and GFH extracts significantly (p < 0.05) decrease of the MDA level compared to the positive control with respective values of 67.09 ± 0.35 and 66.51 ± 1.35 µmol/L.

### Protective effects of extracts on markers involved on oxidative stress

**Fig. 5:**
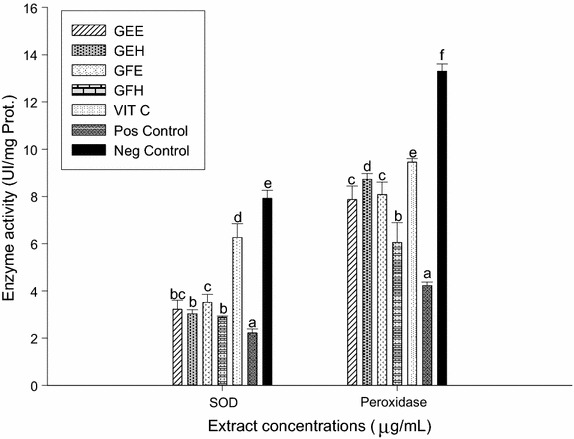
Protective properties of plant extracts: SOD and peroxidase activities. Values are expressed as mean ± SD of three replicates. In the same colon the values affected with *different letter* are significantly different at p < 0.05. *GEE* ethanolic extract of bark of *A. lepidophyllus*, *GFE* ethanolic extract of leaves of *A. lepidophyllus*, *GEH* aqueous ethanol extract of bark of *A. lepidophyllus*, *GFH* aqueous ethanol extract of leaves of *A. lepidophyllus*, *VIT C* vitamin C, *Pos Control* oxidant (positive) control, *Neg* *Control* normal (negative) control.

**Fig. 6:**
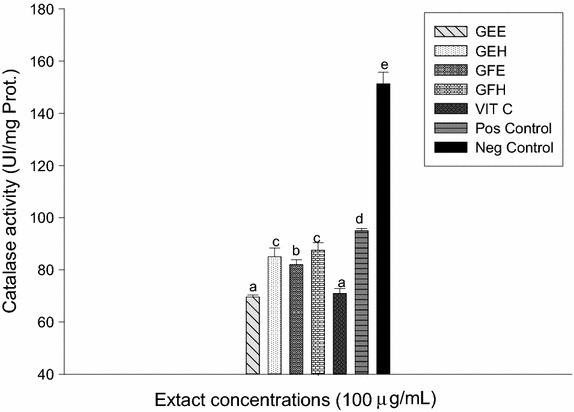
Protective properties of plant extracts: catalase activity. Values are expressed as mean ± SD of three replicates. In the same colon the values affected with *different letter* are significantly different at p < 0.05. *GEE* ethanolic extract of bark of *A. lepidophyllus*, *GFE* ethanolic extract of leaves of *A. lepidophyllus*, *GEH* aqueous ethanol extract of bark of *A. lepidophyllus*, *GFH* aqueous ethanol extract of leaves of *A. lepidophyllus*, *VIT C* vitamin C, *Pos Control* oxidant (positive) control, *Neg Control* normal (negative) control.

**Fig. 7:**
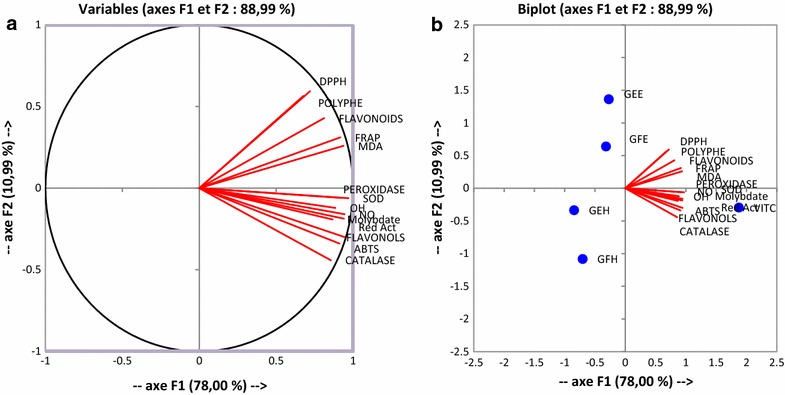
Principal component analysis results on F1XF2 axis of in vitro antioxidant assays corresponding to the tested extracts. *GEE* ethanolic extract of bark of *A. lepidophyllus*, *GFE* ethanolic extract of leaves of *A. lepidophyllus*, *GEH* hydro-ethanolic extract of bark of *A. lepidophyllus*, *GFH* hydro-ethanolic extract of leaves of *A. lepidophyllus*, *VIT C* Vitaùine C, *SOD* SOD activity test, *Catalase* catalase activity test, *Peroxidase* peroxidase activity test, *FLavonols* flavonol assay, *Polyphen* polyphenol assay, *MOLYBDAT* phosphomolybdenum test, *Flavonoids* flavonoid assay, *NO* NO radical scavenging test, *ABTS* ABTS radical scavenging test, *DPPH* DPPH radical scavenging test, *OH* OH radical scavenging test, *Red Act* reductive activity test, *FRAP* FRAP antioxidant test, *MDA* MDA assay.

The results of SOD and peroxidase activities presented in the Fig. 5 showed that the SOD and peroxidase activities of the negative control were significantly higher (p < 0.05) compared to the positive control group (with oxidant). Among tested extracts, GFE had a significantly (p < 0.05) highest SOD activity while GEH demonstrated the highest peroxidase activity. However the GFH had the lowest values both for SOD and peroxidase activities, but these values remained higher than that of vitamin C. These findings showed that the extracts could protect both the SOD and peroxidase activities. The Fig. 6 represented the effects of the extracts on the variation of catalase activity. This result demonstrated that the positive control (with oxidant) showed the lower activity (32.30 ± 1.35 UI/g Prot.) while the negative control (without oxidant) exhibited the highest activity of catalase (151.34 ± 3.59 UI/g Prot.). The catalase activity of all the tested extracts was significantly higher (p < 0.005) compared to the positive control (vitamin C 65.87 ± 5.10 UI/g Prot.). Among the extracts, GFH showed the highest activity. According to these results, the GFH could protect efficiently the catalase activity against oxidant radicals. The correlation between the effects of extract and the markers involved on oxidative stress was confirmed (Fig. 7).

## Discussion

Oxidative systems are essential for the survival of cells. Side effects due of this dependence are the production of free radicals and other reactive oxygen species. They are increasing evidence of the involvement of such species in a variety of normal in vivo regulatory structures [33]. However, when an excess of free radicals is formed, they can overcome protective enzymes and cause destructive cellular effects by oxidizing membrane lipids, cellular proteins, DNA and enzymes, thus shutting down cellular respiration [11]. Several studies demonstrated that plant derived antioxidants may be useful in preventing the deleterious consequences of oxidative stress [34–36]. The importance of natural antioxidant stimulates the research interest on the investigation of protective effects of natural antioxidants from spices [37, 38]. This study demonstrated the free radical scavenging and antioxidant effects of *A. lepidophyllus* a Cameroonian non-timber forest product used as spice in local traditional plates. The ethanol and aqueous ethanol extracts from leaves and barks of *A. lepidophyllus* tested showed significant antioxidant potentials. Among the methods used for the investigation of the antioxidant activities of natural products, the DPPH radical scavenging assay is described, as simple and widely and easy to evaluate antioxidant activity in a relatively short time [39]. The effect of extracts on DPPH˙ radical results for their hydrogen donating ability [39]. DPPH^∙^ is a stable free radical which can accept an electron or hydrogen radical to become a stable diamagnetic molecule. Decrease in absorbance of DPPH^∙^ radical is caused by reaction between antioxidant molecules from investigated extracts and the radical. This results to the scavenging of radical by hydrogen donation which can be observed as a discoloration from purple to yellow. Our results showed an overall DPPH scavenging activity of all the tested samples. Among them, GEE and GFE extracts showed a higher DPPH scavenging potential. Regarding these results, it can be stated that these plant extracts act as proton donors. The scavenging property of these extracts can be attributed to the presence of polyphenols content. Several studies stated that the polyphenols from plants extracts are responsible in a large part for the DPPH scavenging potential [2, 40]. The hydroxyl radical is an extremely reactive free radical formed in biological systems. The hydroxyl radical have been described as a highly damaging species which can cause oxidative damage to DNA, lipids and proteins [41]. Our results demonstrated that the scavenging potential of the extracts increased proportional to the concentration. The extract GEE exhibited a highest OH scavenging power among all the extracts. It can therefore be assumed that these extracts have good antioxidant properties [41]. Nitric oxide (NO) is an important chemical mediator generated by endothelial cells, macrophages, neurons that are involved in the process of several biological processes. An elevated production of NO could lead to several diseases. Oxygen reacts with nitric oxide to generate nitrite and peroxynitrite anions which act as free radicals. Our results showed that extracts can tightly combine to the oxygen and inhibited the generation of the anions. In contrary to what was observed with the OH radical, the GEE extract showed the lowest NO radical scavenging power while GEH and GFH exhibited the highest NO inhibitory potential. The scavenging activities of natural antioxidant from plant origin are attributed to their phenolic composition. Previous research reported that the antioxidant activity of phenolic acids is related to the quantity, number and position of hydroxyl groups in the molecule [42]. ABTS is oxidized by potassium persulfate to its cation radical ABTS^∙+^, which is intensely colored. Our results showed that all the tested samples demonstrated a scavenging potential of the ABTS with varying inhibitory potential with the GEE extract demonstrating the highest ABTS inhibitory power. This result confirmed that the presence of higher flavonoids level in the same extract justified its highest scavenging property on ABTS radical [43]. Studies recommended several testing method for the investigation of antioxidant activity of extracts from natural resources [44]. Reductive power is correlated with the major phenols contents and antioxidant activity of different plant samples. Compounds with high reductive power are electron donors and can reduce the oxidized intermediates of lipid peroxidation process [39]. Regarding our results, the vitamin C, GFH and GEH exhibited highest reductive activity at different concentrations. These results suggest that these two extracts are good electron donors for power reducing activity. Also these extracts can reduce Fe^3+^ to Fe^2+^ in the FRAP assay at pH 3.6 and demonstrated that they have higher antioxidant capacity. This property is always attributed to their high content in polyphenolic compounds [42, 45].

Lipid peroxidation associated with pathogenesis of several illnesses. ROS and RNS are produced in vivo through various biochemical reactions including respiratory chain. These free radicals are the main agents in lipid peroxidation [46]. Our research demonstrated a significant decrease of MDA level in the group tested with extracts compare to the positive control. This result suggests that these extracts have inhibited the oxidation initiation or propagation of lipid peroxidation from polyunsaturated fat of the liver [2]. Mammalian cells possess several defense mechanisms for detoxification of free radicals which include superoxide dismutase (SOD) (EC 1.15.1.1), catalase (EC 1.11.1.6), and peroxidase (EC 1.11.1). Our results showed a significant higher levels of catalase, SOD and peroxidase activities in the negative controls and those treated with extracts compared to the positive controls. The decrease of enzymes activities in the positive control group suggests that the molecules responsible of the antioxidant enzyme activities of that medium were exhausted by the higher concentration of the oxidants [2]. The differences found between the activities of SOD, catalase and peroxidase of the extracts could be attributed either to the qualitative or quantitative difference in the composition of the bioactives molecules present in the extracts. These results suggest that the augmentation of the enzyme activities could be linked to the scavenging potential of the extracts. Similar results were obtained during the investigation of the effect of methanolic extracts of *Solenostemon monostachyus*, *Ipomoea involucrate* and *Carica papaya* seed oil versus glutathione or *Vernonia amygdalina* used on the management of sickle cell anemia disease [47].

## Conclusion

The extracts of *A. lepidophyllus* showed significant free radical scavenging and antioxidant properties. They also exhibited a higher protective potential against some liver markers involved in oxidative stress. The ethanol extract of *A. lepidophyllus* bark demonstrated the most potent activities. Nevertheless, further studies need to be investigated to isolate and characterize the bioactive compounds responsible for these activities and also determine its in vivo protective effects.

## Additional files

Additional file 1: Table S1. Different values of IC_50_ of the plant extracts on the different radicals. Additional file 2: Table S2. Polyphenol, flavonoid and flavonol contents of the different plant extracts. Additional file 3: Table S3. Pearson correlation matrices of *in vitro* antioxidant tests.

## Abbreviations

MDA: malonaldialdehyde
DPPH: 2,2-diphenyl-1-picrylhydrazyl 1,1-diphenyl-2-picrylhydrazyl radical
Vit C: vitamin C
ABTS: 2,2 -azinobis(3-ethylbenzthiazoline)-6-sulfonic acid
BHT: butylated hydroxytoluene
RNS: reactive nitrogen species
ROS: reactive oxygen species
FRAP: ferric reducing antioxidant power
TBA: thiobarbituric acid
H_2_O_2_: hydrogen peroxide
