# Supplementary material for: Afrostyrax lepidophyllus extracts exhibit in vitro free radical scavenging, antioxidant potential and protective properties against liver enzymes ion mediated oxidative damage
Source: BMC Res Notes. 2015 Aug 12;8:344. doi: 10.1186/s13104-015-1304-8 (PMC4534151; doi:10.1186/s13104-015-1304-8)
Supplement: Additional file 1: Table S1. — Different values of IC50 of the plant extracts on the different radicals. [file 13104_2015_1304_MOESM1_ESM.docx]

Table S1: Different values of IC_50_  of the plant extracts on the different radicals

| ***Samples*  *Tests*** | **IC_50_ (µg/mL)** | | | |
| --- | --- | --- | --- | --- |
|  | **OH** | **DPPH** | **NO** | **ABTS** |
| **GFH** | 104.66 ± 7.04^a^ | 195.23 ± 1.94^a^ | 77.67 ± 1.10^a^ | 190.54 ± 1.55^a^ |
| **GEH** | 47.77 ± 1.09^b^ | 185.39 ± 5.14^b^ | 63.08 ± 1.44^b^ | 94.95 ± 2.31^b^ |
| **GFE** | 104.66 ± 6.51^a^ | 82.3 ± 1.11^c^ | 76.66 ± 2.00^c^ | 200.03 ± 1.74^c^ |
| **GEE** | 66.66 ± 2.02^c^ | 64.66 ± 0.02^d^ | 94.73 ± 1.74^d^ | 197.83 ± 1.25^d^ |
| **VIT C** | 27.94 ± 0.04^d^ | 2.55 ± 0.08^e^ | 55.83 ± 0.04^e^ | 78.3 ± 0.01^e^ |

Values are expressed as mean ± SD of three replicates. In the same colon the values affected with different letter are significantly different at p<0.05. Abbreviations: GEE: ethanolic extract of bark of *A. lepidophyllus*, GFE: ethanolic extract of leaves of *A. lepidophyllus*, GEH: aqueous ethanol extract of bark of *A. lepidophyllus*, GFH: aqueous ethanol extract of leaves of *A. lepidophyllus,* VIT C = Vitamin C
