# Supplementary material for: Afrostyrax lepidophyllus extracts exhibit in vitro free radical scavenging, antioxidant potential and protective properties against liver enzymes ion mediated oxidative damage
Source: BMC Res Notes. 2015 Aug 12;8:344. doi: 10.1186/s13104-015-1304-8 (PMC4534151; doi:10.1186/s13104-015-1304-8)
Supplement: Additional file 2: Table S2. — Polyphenol, flavonoid and flavonol contents of the different plant extracts. [file 13104_2015_1304_MOESM2_ESM.docx]

**Table 1: Polyphenol, flavonoid and flavonol contents of the different plant extracts**

| ***Samples*** | **Phytochemicals** | | |
| --- | --- | --- | --- |
|  | **Flavonoids**  (QE/g dried ext) | **Flavonols**  (QE/g dried ext) | **Polyphenols**  (CAE/g dried ext) |
| **GEE** | 12.00 ± 0.14 ^d^ | 2.41 ± 0.75 ^b^ | 35.33 ± 0.29 ^c^ |
| **GFE** | 9.48 ± 0.10 ^c^ | 3.50 ± 0.40 ^c^ | 34.11 ± 0.87 ^c^ |
| **GEH** | 7.10 ± 0.30 ^b^ | 1.55 ± 0.36 ^a^ | 28.21 ± 0.97 ^a^ |
| **GFH** | 1.94 ± 1.05 ^a^ | 2.30 ± 0.04 ^b^ | 30.86 ± 0.87 ^b^ |

Values are expressed as mean ± SD of three replicates. In the same colon the values affected with different letter are significantly different at p<0.05. Abbreviations: GEE: ethanolic extract of bark of *A. lepidophyllus*, GFE: ethanolic extract of leaves of *A. lepidophyllus*, GEH: aqueous ethanol extract of bark of *A. lepidophyllus*, GFH: aqueous ethanol extract of leaves of *A. lepidophyllus,*
