# Supplementary material for: Afrostyrax lepidophyllus extracts exhibit in vitro free radical scavenging, antioxidant potential and protective properties against liver enzymes ion mediated oxidative damage
Source: BMC Res Notes. 2015 Aug 12;8:344. doi: 10.1186/s13104-015-1304-8 (PMC4534151; doi:10.1186/s13104-015-1304-8)
Supplement: Additional file 3: Table S3. — Pearson correlation matrices of in vitro antioxidant tests. [file 13104_2015_1304_MOESM3_ESM.docx]

|  | DPPH | OH | NO | ABTS | Molybdate | Red Act | FLAVONOIDS | FLAVONOLS | POLYPHE | CATALASE | SOD | PEROXIDASE | MDA | FRAP |
| --- | --- | --- | --- | --- | --- | --- | --- | --- | --- | --- | --- | --- | --- | --- |
| DPPH | 1 |  |  |  |  |  |  |  |  |  |  |  |  |  |
| OH | 0,722 | 1 |  |  |  |  |  |  |  |  |  |  |  |  |
| NO | 0,520 | 0,789 | 1 |  |  |  |  |  |  |  |  |  |  |  |
| ABTS | 0,440 | 0,816* | 0,919* | 1 |  |  |  |  |  |  |  |  |  |  |
| Molybdate | 0,654 | 0,958* | 0,753 | 0,865* | 1 |  |  |  |  |  |  |  |  |  |
| Red Act | 0,551 | 0,834* | 0,915* | 0,905* | 0,836* | 1 |  |  |  |  |  |  |  |  |
| FLAVONOIDS | 0,945* | 0,782 | 0,623 | 0,630 | 0,778 | 0,650 | 1 |  |  |  |  |  |  |  |
| FLAVONOLS | 0,523 | 0,894* | 0,919* | 0,977* | 0,912* | 0,923* | 0,681 | 1 |  |  |  |  |  |  |
| POLYPHE | 0,677 | 0,366 | 0,629 | 0,397 | 0,281 | 0,564 | 0,622 | 0,432 | 1 |  |  |  |  |  |
| CATALASE | 0,316 | 0,767 | 0,897* | 0,898* | 0,745 | 0,880* | 0,441 | 0,924* | 0,417 | 1 |  |  |  |  |
| SOD | 0,629 | 0,826* | 0,928* | 0,882* | 0,792 | 0,932* | 0,715 | 0,930* | 0,687 | 0,896* | 1 |  |  |  |
| PEROXIDASE | 0,508 | 0,689 | 0,951* | 0,845* | 0,665 | 0,887* | 0,591 | 0,860* | 0,760 | 0,878* | 0,936* | 1 |  |  |
| MDA | 0,801* | 0,759 | 0,871* | 0,789 | 0,735 | 0,828* | 0,851* | 0,801* | 0,789 | 0,680 | 0,888* | 0,865* | 1 |  |
| FRAP | 0,766 | 0,675 | 0,856* | 0,749 | 0,653 | 0,813* | 0,824* | 0,763 | 0,875* | 0,667 | 0,880* | 0,897* | 0,954* | 1 |
| *: significant values p = 0,050 (bilateral test) | | | | | | | | | | | | | | |

SOD: SOD activity test; Catalase: Catalase activity test; Peroxidase: Peroxidase activity test; FLavonols: Flavonol assay; Polyphen: Polyphenol assay; MOLYBDAT: Phosphomolybdenum test; FLavonols: Flavonol assay; Polyphenol: Polyphenol assay; Flavonoids: Flavonoid assay; NO: NO radical scavenging test; ABTS: ABTS radical scavenging test; DPPH: DPPH radical scavenging test; OH: OH radical scavenging test; Red Act: reductive activity test, Flavonols: Flavonols assay; FRAP: FRAP antioxidant test; MDA: MDA assay;

**Table 3:** Pearson correlation matrices of *in vitro* antioxidant tests
